# Near-Infrared Light-Responsive Nanosystems with Controlled l-Quebrachitol Release and Photothermal Effect for Synergistic Bone Defect Regeneration

**DOI:** 10.34133/bmr.0357

**Published:** 2026-10-30

**Authors:** Zhengkuan Lu, Lingyan Cao, Shuaiting Wu, Ao Zheng, Shuxian Lin, Jiansheng Su

**Affiliations:** ^1^ Shanghai Engineering Research Center of Tooth Restoration and Regeneration & Tongji Research Institute of Stomatology & Department of Prosthodontics, Shanghai Tongji Stomatological Hospital and Dental School, Tongji University, Shanghai 200072, China.; ^2^ Department of Prosthodontics, Shanghai Stomatological Hospital & School of Stomatology, Fudan University; Shanghai Key Laboratory of Craniomaxillofacial Development and Diseases, Fudan University, Shanghai, China.; ^3^ Department of Prosthodontics, Shanghai Ninth People’s Hospital, Shanghai Jiao Tong University School of Medicine; College of Stomatology, Shanghai Jiao Tong University, Shanghai 200011, China.; ^4^ Department of Plastic and Reconstructive Surgery, Shanghai Ninth People’s Hospital, Shanghai 200011, China.

## Abstract

Recent breakthroughs emphasize the growing potential of natural plant extracts in advancing bone regeneration, with l-quebrachitol (LQ) standing out as a promising multifunctional agent. LQ, primarily found in plants from the Sapindaceae, Apocynaceae, and Elaeagnaceae families, has garnered attention for its unique therapeutic properties. In this study, we first utilized the PharmMapper platform to systematically identify pharmacological targets associated with bone formation and angiogenesis for LQ. We then conducted a series of experimental investigations to uncover the underlying signaling pathways, revealing the mechanistic basis for LQ’s action. In order to address the limitations such as short biological half-lives and rapid in vivo degradation, we further integrated LQ into advanced drug delivery systems to enhance its bioavailability and therapeutic potential. Zeolitic imidazolate framework-8 (ZIF-8), a type of metal–organic framework, has a highly porous structure and marked surface area, making it an ideal candidate for drug delivery. We engineered a novel nanocomposite (ZIF-8-pTA-LQ) by surface-modifying ZIF-8 with poly-tannic acid (pTA) and encapsulating LQ within its pore structure. This nanocomposite not only enabled controlled release of LQ but also exhibited mild photothermal properties, rapidly heating to ~45 °C within 4 min under 1.2 W/cm^2^ NIR irradiation and stabilizing below 50 °C within 10 min. The nanosystem facilitated the subsequent activation of the PI3K/Akt signaling pathway, resulting in exceptional performance in regenerating bone defects. This innovative nanoparticle platform, combining controlled drug release with photothermal therapy, presents a novel and highly effective approach to bone tissue repair, offering a promising new avenue for regenerative medicine.

## Introduction

Bone defects caused by disease and accidents seriously affect quality of life; the human body's regenerative capacity is insufficient to restore large bone defects. In clinical treatment, autologous bone grafts do not cause rejection reactions and possess osteoconductive, osteo-inductive, and osteogenic properties. However, when repairing large defects, they remarkably increase the risk of donor site injury and infection. Additionally, cancellous bone constitutes a large proportion of bone grafts and is often partially absorbed or participates in revascularization. For allogeneic bone grafts, although they avoid donor site complications, their sources are scarce and the cost is high. Furthermore, allogeneic bone grafts are nonvascularized and have limited osseointegration capacity with the host bone. They also carry risks of disease transmission and immune rejection. Artificial bone substitute filling is a commonly chosen treatment method today, such as bone powder and periosteum; nevertheless, the surgical procedure has relatively high technical requirements, and the observation period for evaluating efficacy is long [1,2]. To address this clinical problem, there is a clear trend toward applying effective compounds extracted from natural plants in clinical settings.

l-Quebrachitol (LQ) is an inartificial, optically active inositol methoxyl analog discovered in a variety of plants, particularly serum of rubber latex and sea buckthorn, which contain a high concentration of LQ [3]. Increasing focus on LQ arises that, as a novel biomaterial, it has multiple biological activities, including antioxidant, anti-inflammatory, anti-diabetic, anti-cancer, and other activities. d-Pinitol, as an isomer of LQ, performs an inhibitory effect on osteoclastogenesis and presents no function on osteoblastogenesis. Nevertheless, in most cases, isomers perform distinct differences in bioactivity; for instance, the research of Yodthong et al. [4] demonstrated that LQ possesses the capability to promote proliferation, differentiation, and mineralization of the murine pre-osteoblastic cell line MC3T3-E1 by inducing BMP, MAPK, and Wnt/β-catenin signaling pathways possibly.

To approach high loading efficiency as wells as stable and controlled releasing of LQ, metal–organic frameworks (MOFs) have emerged as promising drug delivery carriers in recent years [5]. MOFs are a class of highly ordered crystalline porous coordination polymers that have unique properties and advantages, such as high specific surface area and tunable pore volume, which enable them to adsorb functional molecules on the exterior surfaces or open channels and trap these molecules within the skeleton [6]. In detail, functional molecules can be grafted by a one-pot method or loaded into MOFs through post-synthetic modification strategies via covalent bond. One subclass of MOFs, named as ZIFs, was first investigated to construct an electrochemical biosensor, and ZIF-8 is one kind of them, which consists of a zinc node coordinated with 2-methylimidazole and has been considered as hopeful drug delivery material for its extreme monodispersity, nanoscale size, and specific construction [7,8]. In addition, approaches to achieve controlled release of LQ could be enlightened by several researches, which suggested that near-infrared (NIR) light is capable of promoting the release of drugs loaded on ZIF-8 by impairing or destructing the electrostatic and H-bonding interactions between ZIF-8 and drugs [9,10]. Tannic acid (TA) is a polyphenol extracted from plants and was researched gradually in photothermal treatment (PTT) owing to its negligible cytotoxicity, accessibility, and modifiability [11]; moreover, approaches suggested that TA polymerized into poly-tannic acid (pTA) by coordinating with metal ions such as Fe^3+^ and Cu^2+^, which enhances the ability converting NIR light to heat to a great extent [12,13]. Furthermore, the temperature required for hyperthermia therapy (42 to 45 °C), which is favorable for osteogenesis, could be achieved through the heat generated by PTT [14–17]. The underlying mechanism is that hyperthermia up-regulates heat shock protein (HSP) expression, which is of great importance to the formation and maturation of osteogenesis-related collagen [16–19]. Besides, the combination of effective photodynamic and photothermal treatment generated by NIR, which directly eradicate tumors while maintaining good biocompatibility with normal cells, has made this platform a recent research hotspot [20].

In this study, LQ was firstly investigated on the PharmMapper platform to obtain pharmacological targets related to bone formation and angiogenesis; on this basis, a series of experiments was conducted and the underlying signal pathway was verified. Subsequently, ZIF-8-pTA-LQ nanocomposites composed of ZIF-8, pTA, and LQ were fabricated and its morphology, physicochemical properties, degree of monodispersion, and photothermal performance under 808-nm NIR were characterized. The controlled release of LQ under NIR stimulation was evaluated, followed by the assessment of the effects of mild hyperthermia generated from NIR and released LQ on biocompatibility and osteogenic differentiation ability of bone marrow stem cells (BMSCs). Furthermore, on virtue of preeminent tissue penetration of NIR, a composite of ZIF-8-pTA-LQ and sodium alginate (SA) was constructed and placed in cranial defects of rats to detect the extent of temperature rise on local tissue where NIR irradiated and the impact on vascularization and bone restoration. This research provides a novel approach for the therapy of bone defection using a photothermal way, and it will be more convenient for the treatment of such patients (Fig. 1).

**Fig. 1.**
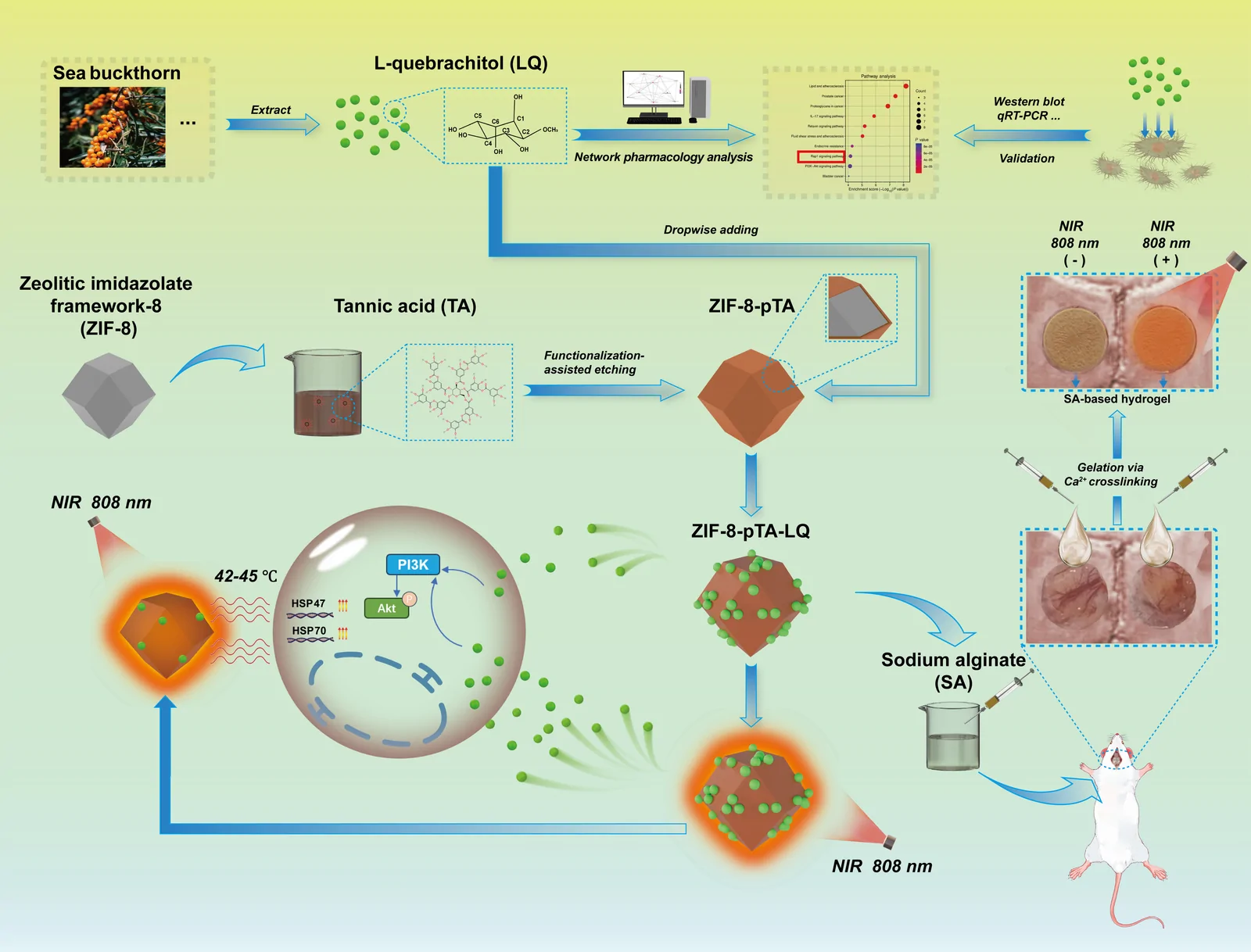
Schematic diagram of preparation of NIR-responsive nanosystem ZIF-8-pTA-LQ and its mechanism of synergistic bone defect regeneration based on photothermal treatment.

## Materials and Methods

### Materials

LQ was purchased from Selleck (USA). Minimum Essential Medium α, 100 U/ml penicillin–streptomycin, and fetal bovine serum (FBS) were purchased from Gibco (USA). Cell Counting Kit-8 (CCK-8), BCIP/NBT Alkaline Phosphatase Color Development Kit, BCA Protein Assay Kit, and Alkaline Phosphatase Assay Kit were purchased from Beyotime Biotech (China). RNAiso Plus and PrimeScript 1st Strand cDNA Synthesis Kit was purchased from TaKaRa Bio (Japan). qPCR SYBR Green Master Mix was purchased from Yeasen (China). Polyvinylidene fluoride (PVDF) membranes were purchased from Millipore (USA). SA, zinc acetate dihydrate, 2-methylimidazole, calcein, and alizarin red S (ARS) were ordered from Sigma-Aldrich (USA). Sprague–Dawley rats (male) were purchased from Shanghai Jihui Laboratory Animal Co. Ltd. (China).

### Network pharmacology analysis method of LQ

#### Obtaining LQ targets

In the method for obtaining LQ targets, it is first necessary to prepare the molecular data of LQ in an structure-data format file (SDF) format. If the molecular data are not in a 3-dimensional conformation, they will be automatically converted by the service. During the experiment, we will use the PharmMapper platform for analysis. This platform utilizes a semi-empirical pharmacophore mapping tool, which requires the generation of multiple molecular conformations. The generation of conformations can be done using an online method through the Cyndi algorithm, a conformation generation tool based on MOEA (Multi-Objective Evolutionary Algorithm). When setting up the Cyndi algorithm, it is necessary to specify the maximum number of conformations generated (Maximum Generated Conformations) and the number of objectives used in MOEA (Number of Objectives used in MOEA). Additionally, we need to select a target database set of human pharmacological targets. Finally, by using a genetic algorithm (Perform GA Match), the pharmacophore map is optimized, and the number of potential targets to be generated (Number of Reserved Matched Targets) is set, thereby obtaining the targets of LQ.

#### Obtaining targets related to osteogenesis and angiogenesis

To obtain targets related to the processes of osteogenesis and angiogenesis, we use the GeneCards (The Human Gene Database) disease gene database. This process first involves entering keywords related to osteogenesis and angiogenesis in the GeneCards database. Subsequently, focus is placed on those genes with a Relevance score greater than or equal to 10, as this score indicates a high degree of relevance of the gene to the search conditions. The Relevance score is a unique scoring system in the GeneCards database, derived from a combination of various bioinformatics tools and data sources, reflecting the relevance of a gene to specific biological processes (BPs) or diseases. By selecting these high-scoring genes, we ensure that the target genes chosen are closely related to the processes of osteogenesis and angiogenesis, providing a valuable set of candidate targets for subsequent research.

#### Selection of intersection targets for LQ in bone formation and angiogenesis treatment

In the study of LQ’s treatment for bone formation and angiogenesis diseases, we utilized R-studio in combination with R version 3.6.3 to select intersection targets, aiming to identify potential therapeutic targets of LQ. Initially, the datasets of LQ’s active targets and the targets related to bone formation and angiogenesis were imported into the R environment. These data were pre-processed to ensure uniform format and accuracy. Subsequently, intersection operations from set theory were applied to identify the common elements between the 2 datasets, which are the targets shared by LQ in the processes of bone formation and angiogenesis. These intersection targets are considered key action points of LQ in the treatment of bone formation and angiogenesis diseases.

#### Enrichment analysis of therapeutic targets

In the enrichment analysis of therapeutic targets for the treatment of bone formation and angiogenesis diseases with LQ, we used Rstudio with R version 3.6.3 and loaded specific bioinformatics analysis packages such as “org.Hs.eg.db”, “enrichplot”, and “clusterProfiler”. These tools were used to perform Kyoto Encyclopedia of Genes and Genomes (KEGG) pathway analysis and Gene Ontology (GO) enrichment analysis. Firstly, the list of selected therapeutic targets was input into these analysis tools to investigate their enrichment in BPs, molecular functions (MFs), and cellular components (CCs), as well as their roles in various biological pathways. This step is instrumental in understanding the potential mechanisms of these targets in the processes of bone formation and angiogenesis. Subsequently, the “ggplot2” package was used to visualize the enrichment results, creating intuitive and informative charts. These charts not only display the distribution of targets in biological pathways and functions but also help identify key pathways and functional categories that may play a central role in the processes of bone formation and angiogenesis, thereby providing important biological insights for subsequent experimental research and drug development.

### Verification of the functional mechanism of LQ

#### Cell culture

Rat BMSCs were isolated from 3-week-old Sprague–Dawley rats. In detail, BMSCs collected from femurs and tibias were cultured in minimum essential medium α (α-MEM) culture medium, mixed with 10% FBS and 1% penicillin–streptomycin. The cells were incubated in 10-cm culture dishes under the humid atmosphere with 5% CO_2_ at 37 °C (Heracell 150i, Thermo Fisher Scientific, USA). The culture medium was refreshed every 2 to 3 d, and the third passage BMSCs were used in the following cell experiments.

#### Cell viability

BMSCs of the third passage were seeded in a 96-well plate at a density of 1 × 10^4^ cells per well. After growing overnight, cells were treated with LQ at concentrations of 0, 0.2, 2, 20, 200, and 2,000 μg/ml for 1 and 3 d. At abovementioned time points, the medium was discarded and the growth medium containing 10% CCK-8 Kit solution was added in each well, and the plate with samples was incubated at 37 °C for 2 h. Finaly, the absorbance was measured at 450 nm using a microplate reader (Safire, TECAN, Switzerland).

#### Alkaline phosphatase staining and activity measurement

BMSCs were cultured in a 24-well plate at a density of 1 × 10^4^ cells per well overnight. While the cell confluence achieved 90%, original growth medium was discarded and an osteogenic inductive medium with LQ at concentrations of 0, 2, 20, and 200 μg/ml was added. After osteogenic culture for 3 d, the samples were fixed with 4% paraformaldehyde for 20 min followed by fully rinsing with phosphate-buffered saline (PBS). BCIP/NBT Alkaline Phosphatase Staining Kit was added and fully covered the surface of sample. Samples were incubated for additional 30 min and observed using an optical microscope. Alkaline phosphatase (ALP) activity was measured with BCA Protein Assay Kit and Alkaline Phosphatase Assay Kit according to the company’s experimental procedures.

#### Quantitative real-time polymerase chain reaction

BMSCs were cultured in a 24-well plate at a density of 1 × 10^4^ cells per well overnight. While the cell confluence achieved 90%, original growth medium was discarded and an osteogenic inductive medium with LQ at concentrations of 0, 2, 20, and 200 μg/ml was added. After osteogenic culture for 3 d, total RNA was extracted with RNAiso Plus. Subsequently, cDNA was obtained through reverse transcription with the PrimeScript1st Strand cDNA Synthesis Kit. Quantitative real-time polymerase chain reaction (qRT-PCR) was performed using a real-time fluorescence quantitative PCR system (LightCycler 480, Roche, Switzerland) with qPCR SYBR Green Master Mix. The expression levels of osteogenesis-related [ALP, osteocalcin (OCN), osteopontin (OPN), runt-related transcription factor 2 (RUNX-2), collagen-1 (COL-1)] and angiogenesis-related [vascular endothelial growth factor (VEGF)] genes were quantitatively determined by the ΔΔ*C*_t_ method. The primer sequences were shown in Table S1.

#### Western blot and qRT-PCR

BMSCs were cultured in α-MEM containing LQ. After stimulation for 0, 30, 60 and 90 min, the protein expression levels of p-PI3K (phosphatidylinositol 3-kinase), PI3K, p-Akt, and Akt were detected by Western blotting. Briefly, equal amounts of proteins collected from BMSCs were loaded on 10% sodium dodecyl sulfate–polyacrylamide and transferred onto preactivated PVDF membranes. The membrane was blocked with 5% skim milk solution for 2 h at room temperature and subsequently incubated with primary antibodies targeting p-PI3K, PI3K, p-Akt, Akt, and β-actin (Proteintech, USA) at 4 °C overnight. Finaly, after incubation with secondary antibodies for 1 h, the protein bands were visualized using an enhanced chemiluminescence detection system with exposure to FluorChem HD2 (Cell Biosciences Inc., Santa Clara, CA, USA). Furthermore, BMSCs were cultured in medium containing 10 × 10^−6^ M LY294002 (LY) (MCE, USA) or LQ. BMSCs cultured in medium without LY and LQ were used as the control group, the proteins of p-PI3K, PI3K, p-Akt, and Akt were detected with Western blot assay; in addition, mRNA was extracted from another portion of BMSCs, and the expression levels of PI3K/Akt signaling factors [glycogen synthase kinase-3β (GSK-3β) and β-catenin], osteogenic genes (RUNX2, OCN, and BMP-2), and angiogenesis-related gene (VEGF) were also detected to support potential pathways of LQ’s function.

### Synthesis and characterization of ZIF-8-pTA-LQ

#### Synthesis of ZIF-8

Initially, 18.7 g of zinc acetate dihydrate was dissolved in 200 ml of deionized water and stirred for 1 h to completely dissolve zinc acetate dihydrate to obtain zinc precursor solution. 2-Methylimidazole (56 g) was added into 300 ml of deionized water and stirred for 1 h with a magnetic stirring bar to acquire a completely dissolved organic linker solution. Subsequently, the zinc precursor solution and the organic linker solution were mixed with a stirring rate of 400 rpm for 10 min, and the mixture was aged for 24 h at room temperature covered with layers of parafilm. The obtained white solution was centrifuged at 39,191 relative centrifugal force (r.c.f.) for 20 min, following by the washing process for several times with deionized water. White ZIF-8 powder was prepared for subsequent use after drying at 80 °C in air-forced oven.

#### Synthesis of ZIF-8-pTA

TA (1.25 g) was dissolved in 50 ml of deionized water upon sonication for 60 min, and a clear brownish TA solution was obtained. Five milliliters of ZIF-8 (0.1 g/ml) was added into 45 ml of deionized water with stirring for 40 min. The TA solution was slowly transferred into the ZIF-8 solution while stirring for 3 h at 400 rpm. The mixed solution was centrifuged at 3,273 r.c.f. for 15 min and washed with deionized water and methanol in turn, and the process was repeated for several times. Finally, ZIF-8-pTA, which appears as a pale brown powder, was obtained after drying at 80 °C.

#### Synthesis of ZIF-8-pTA-LQ

Firstly, 2.0 mg of LQ was dissolved in 100 μl of dimethyl sulfoxide to prepare the LQ solution. ZIF-8-pTA (5 mg) nanoparticles are dispersed into the prepared LQ solution and stirred gently for 3 h. After the mixture was left to stand for 12 h, it was dried to obtain the ZIF-8-pTA-LQ nanoparticles.

#### Characterization of ZIF-8-pTA-LQ

##### Scanning electron microscopy

A small amount of ZIF-8 and ZIF-8-pTA powder was dissolved in alcohol solution, fully shaken and mixed, then dripped onto the slide, left for 30 min, and then sent to scanning electron microscopy (SEM) (Sigma 300, ZEISS, Germany) for detection after the ethanol evaporated. The nanopowder was placed on the SEM copper plate, and after gold spraying, the copper plate with the sample was placed on the SEM shelf. At an operating voltage of 3 kV, the accelerated electrons focus on the surface of the particle and stimulate it to produce a physical signal, which is accepted and transformed to present the morphology of the material surface.

##### Transmission electron microscopy

The ZIF-8 and ZIF-8-pTA powder were added into the alcohol solution, mixed by ultrasonic shock, and then sent to the sample. During the test, a small amount of samples were dipped and placed on 400-mesh ultrathin carbon film copper net, and then placed on the stage for observation after the solution evaporated. By adjusting the field of view and voltage, the accelerated and concentrated electron beam is projected onto the sample to obtain the transmission electron microscopy (TEM) (F200X G2, FEI Talos, USA) image of ZIF-8. Energy-dispersive spectroscopy (EDS) was also performed to detect the content of the elements that constitute ZIF-8 and ZIF-8-pTA.

##### Fourier transform infrared test

The qualitative analysis of ZIF-8, pTA, ZIF-8-pTA, LQ, and ZIF-8-pTA-LQ was carried out by Fourier transform infrared (FTIR) (Nicolet iS20, Thermo Fisher Scientific, USA). The above powder was placed on a potassium bromide disk to obtain an FTIR spectrum in the range of 400 to 4,000 cm^−1^ on an FTIR spectrometer.

##### Thermogravimetric analysis

Thermogravimetric analysis (TGA) was performed utilizing instrument STA 449 F3 (Netszch, Germany) at a fixed heating rate (10 °C/min) in an argon atmosphere ranging from room temperature to 1,100 °C.

### Cytotoxicity of ZIF-8-pTA-LQ

BMSCs of the third passage were seeded in a 96-well plate at a density of 1 × 10^4^ cells per well. After growing overnight, cells were treated with ZIF-8-pTA and ZIF-8-pTA-LQ at concentrations of 0, 18.75, 37.5, 75, and 150 μg/ml for 1, 3, and 5 d. At abovementioned time points, the medium was discarded and the growth medium containing 10% CCK-8 Kit solution was added in each well. The plate with samples was incubated at 37 °C for 2 h. Finaly, the absorbance was measured at 450 nm using a microplate reader (Safire, TECAN, Switzerland).

### Photothermal properties of ZIF-8-pTA-LQ

The photothermal properties of ZIF-8-pTA-LQ were assessed using an 808-nm NIR laser. Firstly, 150 μl of PBS suspension with gradient concentrations of ZIF-8-pTA-LQ (0, 37.5, and 75 μg/ml) was prepared in Eppendorf tubes. Then, the suspension was irradiated vertically at a height of 10 cm with an 808-nm NIR laser (1.2 W/cm^2^) for 600 s. The process of temperature altering of each sample was recorded with an infrared thermal imager (Fotric 280, China). Additionally, the photothermal stability of ZIF-8-pTA-LQ was evaluated for 3 cycles of the heating–cooling process.

### Release behavior of ZIF-8-pTA-LQ with stimulation of NIR

The release process of LQ from ZIF-8-pTA-LQ was tested. ZIF-8-pTA-LQ (1 mg/ml) was resuspended in 1 ml of PBS (pH 5 or 7.4). The samples were placed in a shaker under 100 rpm stirring and 37 °C, and the irradiation group was treated with 808-nm NIR laser for 10 min per day. After centrifugation, 500 μl of solution was collected at predetermined time intervals (1, 2, 4, 8, 12, 24, 48, 96, 168, and 336 h). Thereafter, 500 μl of appropriate buffer was added back. The released LQ was quantitated by high-performance liquid chromatography.

### In vitro osteogenic differentiation assessment

#### Cell proliferation

BMSCs were cultured in 96-well plate at a density of 1 × 10^3^ cells per well overnight. Next, the ZIF-8-pTA and ZIF-8-pTA-LQ dispersions were diluted with the growth medium to a concentration of 75 μg/ml, and cells cultured in the medium without both nanoparticles served as the control group. Simultaneously, samples were divided into 2 additional groups: one with NIR laser irradiation and one without. At days 1, 3, and 5, cell viability of each group was determined as CCK-8 way.

#### ALP staining and activity measurement

BMSCs were cultured in a 24-well plate at a density of 1 × 10^4^ cells per well overnight. While the cell confluence achieved 90%, original growth medium was discarded and an osteogenic inductive medium with or without ZIF-8-pTA or ZIF-8-pTA-LQ was added. Half of the samples were treated with periodical NIR laser for 6 min every day. After osteogenic culture for 7 d, the samples were fixed with 4% paraformaldehyde for 20 min followed by fully rinsing with PBS. The BCIP/NBT Alkaline Phosphatase Staining Kit was added and fully covered the surface of the sample. Samples were incubated for additional 30 min and observed using an optical microscope. ALP activity was measured with BCA Protein Assay Kit and Alkaline Phosphatase Assay Kit according to the company’s experimental procedures.

#### ARS staining

BMSCs were cultured in a 24-well plate at a density of 1 × 10^4^ cells per well overnight. While the cell confluence achieved 90%, original growth medium was discarded and an osteogenic inductive medium with or without ZIF-8-pTA or ZIF-8-pTA-LQ was added. Half of the samples were treated with periodical NIR laser for 6 min every day. After osteogenic culture for 21 d, the samples were fixed with ethyl alcohol for 30 min and stained with 1% alizarin red dye. Samples were observed and photographed under an inverted microscope. Subsequently 10% cetylpyridinium chloride was added into wells that stained with ARS, while alizarin red was dissolved, the supernatant was collected, and the absorbance at 590-nm wavelength was detected to evaluate the semiquantitative value of ARS of samples.

#### qRT-PCR

The process of cell culture is the same as abovementioned procedures, and BMSCs were cultured in a 6-well plate at a density of 1 × 10^6^ per well. Total RNA was extracted with RNAiso Plus. Subsequently, cDNA was obtained through reverse transcription with the PrimeScript 1st Strand cDNA Synthesis Kit. The expression levels of osteogenic differentiation-related genes involving RUNX-2, COL-1, bone morphogenetic protein-2 (BMP-2), OPN, and OCN were detected. Secondly, BMSCs cultured in a 6-well plate were divided into 3 groups adopting different processing methods (con, ZIF-8-pTA, and NIR-ZIF-8-pTA), and the expression levels of HSP-related genes containing HSP47, HSP70A, HSP70B, and HSP70C were also detected. The gene expression levels were quantitatively determined by the ΔΔ*C*_t_ method, with β-actin serving as internal reference. The primer sequences were shown in Table S1.

### In vivo cranial defect healing assay

#### Cranial defect construction

All experimental procedures were approved by and performed in accordance with the standards of the Animal Welfare Committees of Tongji University in Shanghai, China (number: TJI-HB-LAC-2025-155). A total of 45 six-week-old male Sprague–Dawley rats weighing 200 to 250 g were randomly divided into 3 groups: blank/SA group, ZIF-8-pTA group. and ZIF-8-pTA-LQ group. Each group included 15 rats. The cranial defects were prepared as previously reported. In detail, after anesthetization and disinfection, the 2 circular defects in the left and right sides of the sagittal suture were prepared with 5-mm-diameter trephine. Depending on the group, 30 μl of SA containing different components was injected into the bone defects; especially, the left cranial defects of rats in the blank/SA group were not filled with material as blank control, and the right side of defects was injected with pure SA. Additionally, except for blank/SA group, the right cranial defects were irradiated with 808-nm NIR laser (1.2 W/cm^2^) for 6 min every 2 d, and the process of temperature increase was recorded for in vivo photothermal assay.

#### Sequential fluorescence labeling

Sequential fluorescence labeling was performed on one-third of rats in each group to assess the level of new bone formation and mineralization. At weeks 6 and 9 after surgery, a series of fluorescent agents were injected intraperitoneally: 20 mg/kg calcein and 30 mg/kg alizarin red S. The rats were sacrificed on week 12, and the skulls were extracted. The hard tissue was sliced using Leica SP1600 (Germany) after dehydration and embedding. Finally, the sections were surveyed with a confocal laser scanning microscope (CLSM).

#### Micro-CT detection of new vessel and new bone formation

Another one-third of rats were sacrificed and perfused with Microfil perfusion (Flow Tech Inc., USA) at 4 weeks post-operation. Samples were scanned by micro-computed tomography (CT) after decalcification to evaluate the level of vascularization in the cranial defects. The remaining rats were sacrificed at 8 weeks after surgery, and the skull specimens were scanned using micro-CT to assess the level of new bone formation. The new bone volume and number of new trabeculae were also evaluated.

#### Histological and histomorphometric observation

After micro-CT evaluation, the samples were decalcified, embedded in paraffin, and sliced into 4-μm sections. A subset of sections of 4 weeks was used for immunohistochemical staining [tumor necrosis factor-α (TNF-α)] to assess the inflammatory response within the tissue, and the major organs harvested from the rats were subjected to hematoxylin and eosin (H&E) staining to assess the potential systemic toxicity of the injected nanoparticles. Besides, part of section specimens was prepared for immunofluorescence staining. Briefly, the slices were incubated with primary antibodies for CD31 and OPN (Abcam, USA), followed by fluorescently labeled secondary antibodies (Abcam, USA) and 4′,6-diamidino-2-phenylindole (DAPI), and finally observed using CLSM. Certain sections of 8-week specimens were stained with H&E and Masson’s trichrome. To further validate the mechanism of function of this nanosystem in vivo, selected 4-week samples underwent immunohistochemical staining (HSP47 and p-Akt).

### Statistical analysis

All quantitative data are the average of 3 independent experiments and are presented as the mean ± SD. All data were analyzed by Origin 9.0. *P* < 0.05 was considered a significant difference. After testing the normality, statistical analysis was performed using a one-way analysis of variance followed by the least significant difference test.

## Results and Discussion

### LQ-disease intersection target construction results

In our research on the therapeutic effects of LQ and its relationship with bone formation and angiogenesis diseases, we made several important discoveries. Initially, through predictions from the PharmMapper database, we identified 225 potential targets of LQ. Subsequently, in the GeneCards database, based on the set selection criteria (i.e., a relevance score of 10 or higher), we found 389 target genes related to bone formation and angiogenesis. Through intersection analysis, we determined 36 common targets between LQ and bone formation and angiogenesis diseases, including key genes such as BMP2, ESR1, MMP13, MAPK1, TTR, CASP3, PPARG, EGFR, MAPK14, PTPN11, KDR, and SRC. The identification of these targets provided preliminary clues about the mechanisms through which LQ may act in the treatment of bone formation and angiogenesis, laying the foundation for future experimental validation and drug development research (Fig. 2A).

**Fig. 2.**
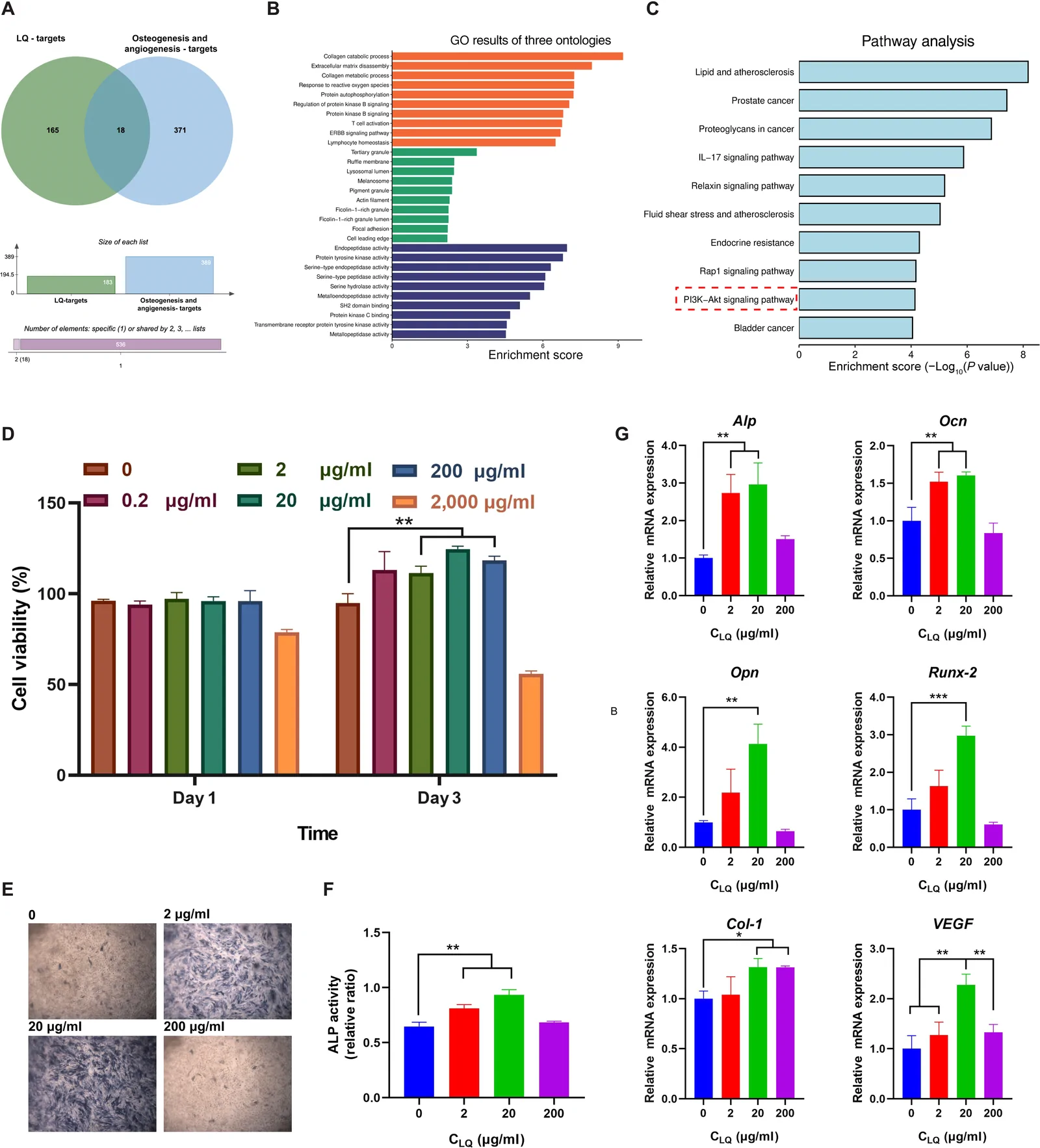
Network pharmacology analysis and performance in osteogenesis and angiogenesis on BMSCs of LQ. (A) Intersection graph of LQ targets with bone formation and angiogenesis. (B) Graph of the results of GO enrichment analysis for communicative targets. (C) Graph of the results of KEGG enrichment analysis for communicative targets. (D) Cell viability of BMSCs after treatment with gradient concentration of LQ. The data represented means ± SD (*n* = 3). (E) ALP staining of BMSCs after treatment with gradient concentration of LQ. (F) ALP activity assay of BMSCs after treatment with gradient concentration of LQ. The data represented means ± SD (*n* = 3). (G) Relative mRNA expression associated with osteogenesis and angiogenesis (including ALP, OCN, OPN, RUNX2, COL-1, and VEGF) after treatment gradient concentration of LQ. The data represented means ± SD (*n* = 3). *P < 0.05, **P < 0.01, ***P < 0.001.

### Results of the enrichment analysis of intersection targets

In the results of the functional enrichment analysis of intersection targets between LQ and bone formation and angiogenesis diseases, we discovered several key insights. As shown in Fig. 2B, through GO enrichment analysis, we identified 38 entries related to CCs, 134 entries related to MFs, and 1,716 entries related to BPs. These results covered multiple levels from cellular structures to molecular activities, to entire BPs, providing a comprehensive understanding of the functions of intersection targets. Furthermore, as shown in Fig. 2C, through enrichment analysis in the KEGG, we identified 131 related biological pathways. These pathways include cancer-related glycoprotein pathways, MAPK signaling pathway, lipid metabolism and arteriosclerosis, relaxin signaling pathway, PI3K-Akt signaling pathway, endocrine resistance, Ras signaling pathway, and advanced glycation end products–receptor for advanced glycation end products (AGE-RAGE) signaling pathway in diabetic complications. These rich enrichment analysis results not only revealed the roles of LQ intersection targets with bone formation and angiogenesis at multiple biological levels but also provided strong bioinformatics support for a deeper understanding of the potential mechanisms of LQ in the treatment of bone formation and angiogenesis.

### In vitro mechanism research of LQ

As one of the abovementioned intersection targets of the functional enrichment analysis between LQ and bone formation and angiogenesis, the PI3K/Akt signaling pathway was chosen for experimental validation because of its critical role in biological activity procedures, especially in proliferation and osteogenic differentiation of osteoblast and bone formation [21–23], consistent with the aims of this research. Firstly, the optimal concentration of LQ for promoting both osteogenic and angiogenic differentiation of BMSCs was screened using CCK-8 assay (Fig. 2D), ALP staining, and qRT-PCR experiments, as Fig. 2E and F depicted. When the concentration of LQ was 20 μg/ml, the results demonstrated the most intense ALP staining and the highest ALP activity. For the crucial markers in the bone formation process containing osteogenesis-related genes (ALP, BSP, OCN, OPN, and RUNX-2) and angiogenesis-related genes (VEGF) [24,25], relatively high mRNA expression levels were also observed (Fig. 2G). Subsequently, Western blot analysis and qRT-PCR were performed to further verify the correlation between LQ and PI3K/Akt signaling pathway. In PI3K/Akt signaling pathway, while being activated, PI3K could assist phosphorylating Akt, and GSK-3β, which is one of the downstream protein targets, could be phosphorylated and inhibited by phosphorylated Akt (p-Akt). Finally, more β-catenin could enter the nucleus and activate the expression of related genes [26–30]. Figure 3A illustrates that levels of p-PI3K and the phosphorylation of Akt were enhanced after the addition of LQ at 60 and 90 min. A PI3K-specific inhibitor, LY, was employed, as shown in Fig. 3B. The addition of LY could reverse the up-regulation of the protein levels of p-PI3K and p-Akt, the levels of mRNA of GSK-3β and β-catenin aligned with experimental predictions (Fig. 3C), and the ALP staining and osteogenesis-related genes (OCN, BMP-2, and RUNX-2) increased upon addition of LQ, while supplementation with LY correspondingly reduces the expression levels. Taken together, these results demonstrated that the PI3K/Akt signaling pathway could be activated in BMSCs driven by LQ, and LQ could stimulate osteogenic differentiation of BMSCs and up-regulate the mRNA expression of VEGF.

**Fig. 3.**
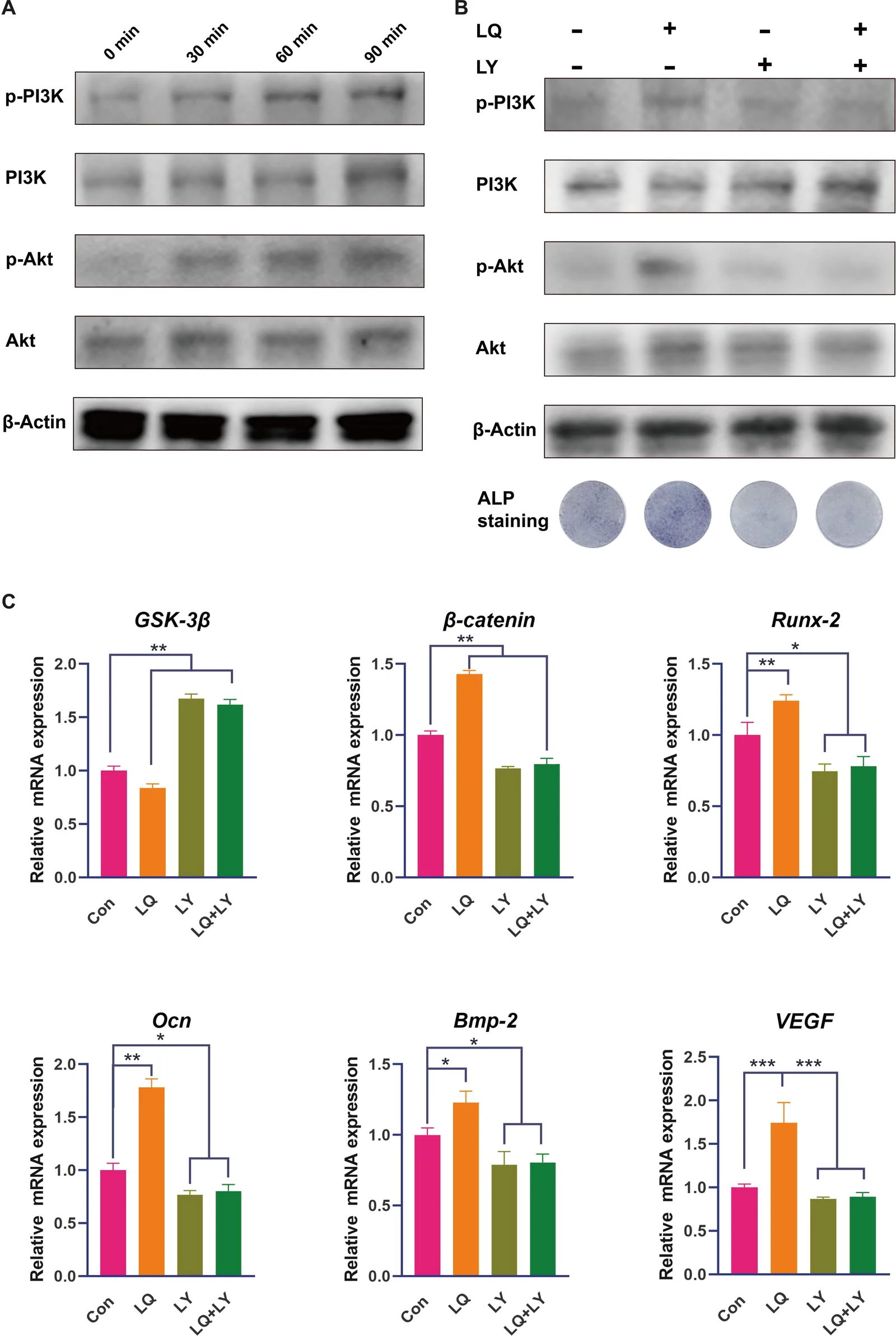
Verification of the functional mechanism of LQ. (A) Western blot results of p-PI3K, PI3K, p-Akt, aand Akt in BMSCs after treatment with LQ in a time-dependent gradient manner. (B) Western blot results of p-PI3K, PI3K, p-Akt, Akt, and ALP staining after treatment with or without LQ or LY for 3 d. (C) Relative mRNA expression associated with osteogenesis and PI3K/Akt signaling pathway (including GSK-3β, β-catenin, RUNX2, OCN, BMP-2, and VEGF) after treatment with or without LQ or LY. The data represented means ± SD (*n* = 3). *P < 0.05, **P < 0.01, ***P < 0.001.

### Preparation and characterization of ZIF-8-pTA-LQ

The microstructures of ZIF-8 and ZIF-8-pTA were observed and analyzed by means of SEM and TEM. As shown in Fig. 4A to H, the ZIF-8 nanoparticles exhibited a typical hexagonal shape in both images and had a uniform diameter of 58.24 ± 9.04 nm. In comparison, the surface of ZIF-8-pTA was rougher, with a distinct shell layer covering the nanoparticles. In addition, EDS mapping showed a uniform distribution of C, O, N, and Zn in ZIF-8 (Fig. 4B). Besides, the augment of C and O content shown in Fig. 4D, which is also presented in Fig. 4C with denser brightness around the shell structure, confirms the successful modification of TA on ZIF-8. FTIR was conducted to characterize the chemical functional group of nanoparticles. As shown in Fig. 4I, the characteristic peaks at 3,415 and 1,679 cm^−1^, which correspond to the stretching vibration of O–H and C=O, were also contained in the spectrum of ZIF-8-pTA and ZIF-8-pTA-LQ. The typical absorption of ZIF-8 at 1,140 and 420 cm^−1^ was assigned to the stretching vibration of C–N and Zn–N. Additionally, the bending vibration of the imidazole ring results in the absorption at 765 and 684 cm^−1^, and these peaks could also be distinctly observed in the ZIF-8-pTA and ZIF-8-pTA-LQ groups. In contrast with the spectrum of LQ, the typical peaks at 909 and 863 cm^−1^ was found in the ZIF-8-pTA-LQ group [31–36]. Combining the above results, this analysis indicated that ZIF-8-pTA and ZIF-8-pTA-LQ were successfully synthesized. TGA is a technique that investigates the thermal stability and composition of a substance by measuring its mass changes under controlled temperature programs. As described in Fig. 4J, when the temperature rises to 200 °C, the mass of pTA began to decline rapidly and completely decomposed at approximately 330 °C. The quality of LQ decreased mildly within 600 °C and promptly decomposed with the remaining mass of about 38%. As for ZIF-8, at the temperature of 789 °C, the mass–temperature curve presented obvious variation, the degradation tendency decelerated, and a marked peak of derivative thermogravimetric (DTG) also emerged. Moreover, this characteristic could be clearly observed in ZIF-8-pTA and ZIF-8-pTA-LQ. Especially, the residual mass of ZIF-8 (34.99%) is slightly more than that of ZIF-8-pTA (33.35%), which is consistent with the TGA performance of pTA. Additionally, the typical peak in the DTG graph of LQ, which was attributed to the quick decomposition above the temperature of 614 °C, was correspondingly detected in the ZIF-8-pTA and ZIF-8-pTA-LQ groups. Collectively, these results confirmed the successful fabrication of ZIF-8-pTA and ZIF-8-pTA-LQ.

**Fig. 4.**
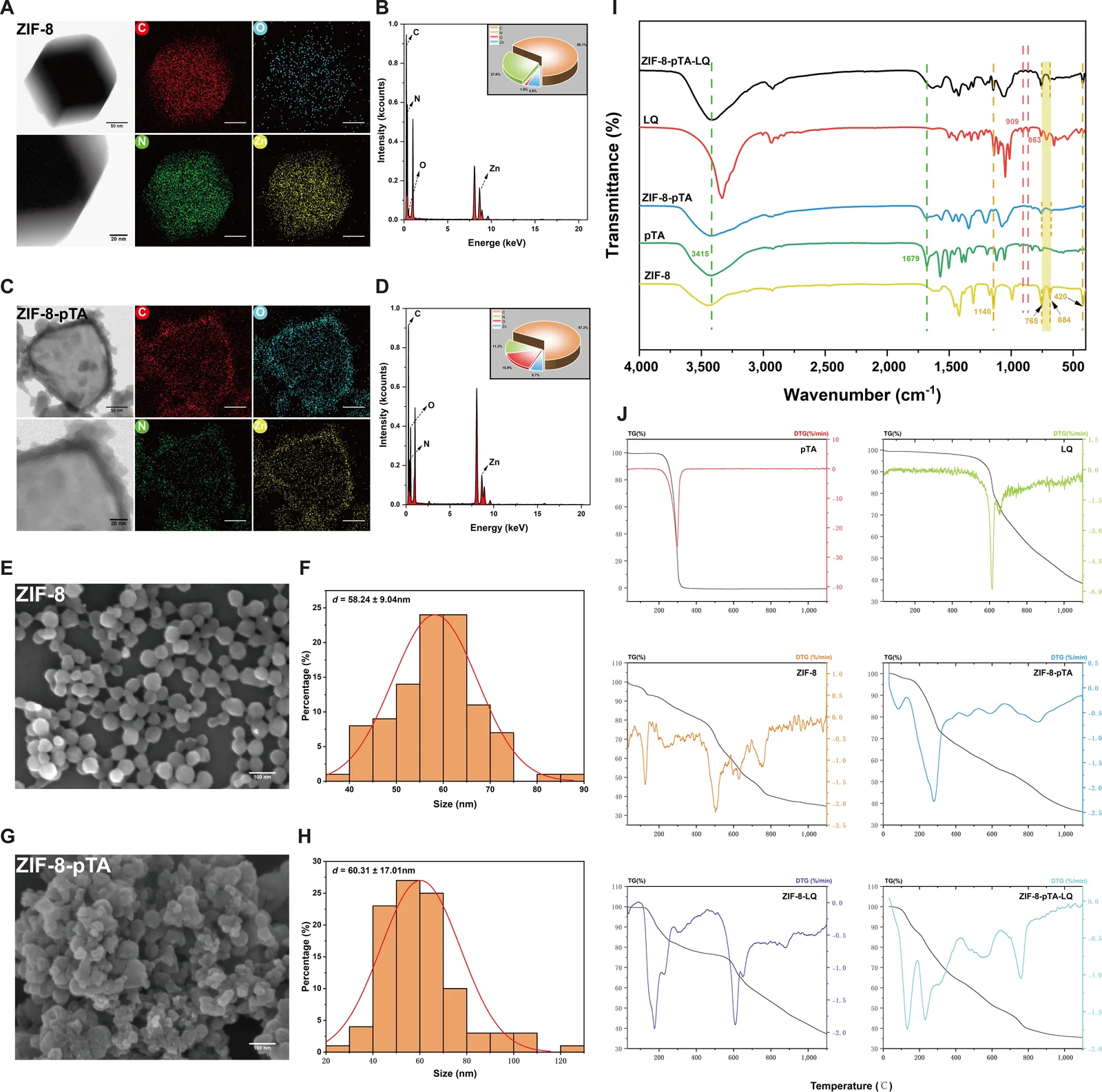
Preparation and characterization of ZIF-8 and ZIF-8-pTA. (A) TEM and element mapping (C, N, O, Zn) images of ZIF-8 nanoparticles (scale bar, 50 nm). (B) EDS elemental mapping of the ZIF-8 and pie chart statistics of C, N, O, and Zn content. (C) TEM and element mapping (C, N, O, and Zn) images of ZIF-8-pTA nanoparticles (scale bar, 50 nm). (D) EDS elemental mapping of the ZIF-8-pTA and pie chart statistics of C, N, O, and Zn content. (E) SEM images of ZIF-8. (F) ZIF-8 particle size distribution. (G) SEM images of ZIF-8-pTA. (H) ZIF-8-pTA particle size distribution. (I) FTIR spectra of ZIF-8, pTA, ZIF-8-pTA, LQ, and ZIF-8-pTA-LQ. (J) TGA and DTG curves of pTA, LQ, ZIF-8, ZIF-8-pTA, ZIF-8-LQ, and ZIF-8-pTA-LQ.

### Biocompatibility of ZIF-8-pTA-LQ

A series of gradient concentrations of ZIF-8-pTA and ZIF-8-pTA-LQ were set to evaluate the toxicity and screen the optimum function concentration in the subsequent experiments. As shown in Figs. S1 and S2, a obvious decrease of cell viability took place, while the concentration of ZIF-8-pTA and ZIF-8-pTA-LQ was 150 μg/ml on days 1, 3, and 5. In consideration of this result, concentrations of 37.5 and 75 μg/ml were used in the following assays.

### Photothermal property of ZIF-8-pTA-LQ

The photothermal property was examined with ZIF-8-pTA-LQ concentrations of 0, 37.5, and 75 μg/ml, which were screened by the abovementioned biocompatibility detection. Initially, on the basis of previous PTT-related literature and researches, the most commonly used power 1.2 W/cm^2^ was adopted. As shown in Fig. 5A, the temperature of ZIF-8-pTA-LQ suspension at concentrations of 37.5 and 75 μg/ml was clearly higher than that of PBS after 10 min of NIR laser irradiation and performed a concentration-dependent appearance. Briefly, 75 μg/ml of ZIF-8-pTA-LQ in PBS could generate a temperature increase of approximately 20 °C, whereas the temperature rise in PBS is merely around 5 °C. Furthermore, the temperature performed a rapid increase within 4 min in the 75 μg/ml group, and the increase rate of temperature slowed down in the following 4 to 10 min and finally stabilized at approximately 45 °C (Fig. 5B), which maintained biosafety requirements while coinciding with the concept of hyperthermia therapy [37,38]. Additionally, the photothermal stability of ZIF-8-pTA-LQ was evaluated. During the 3 stimulation cycles, results performed nearly identical temperature changing curves and maximum temperature points (Fig. 5C), which suggested that ZIF-8-pTA-LQ was capable of maintaining the photothermal conversion efficiency for reduplicative PTT.

**Fig. 5.**
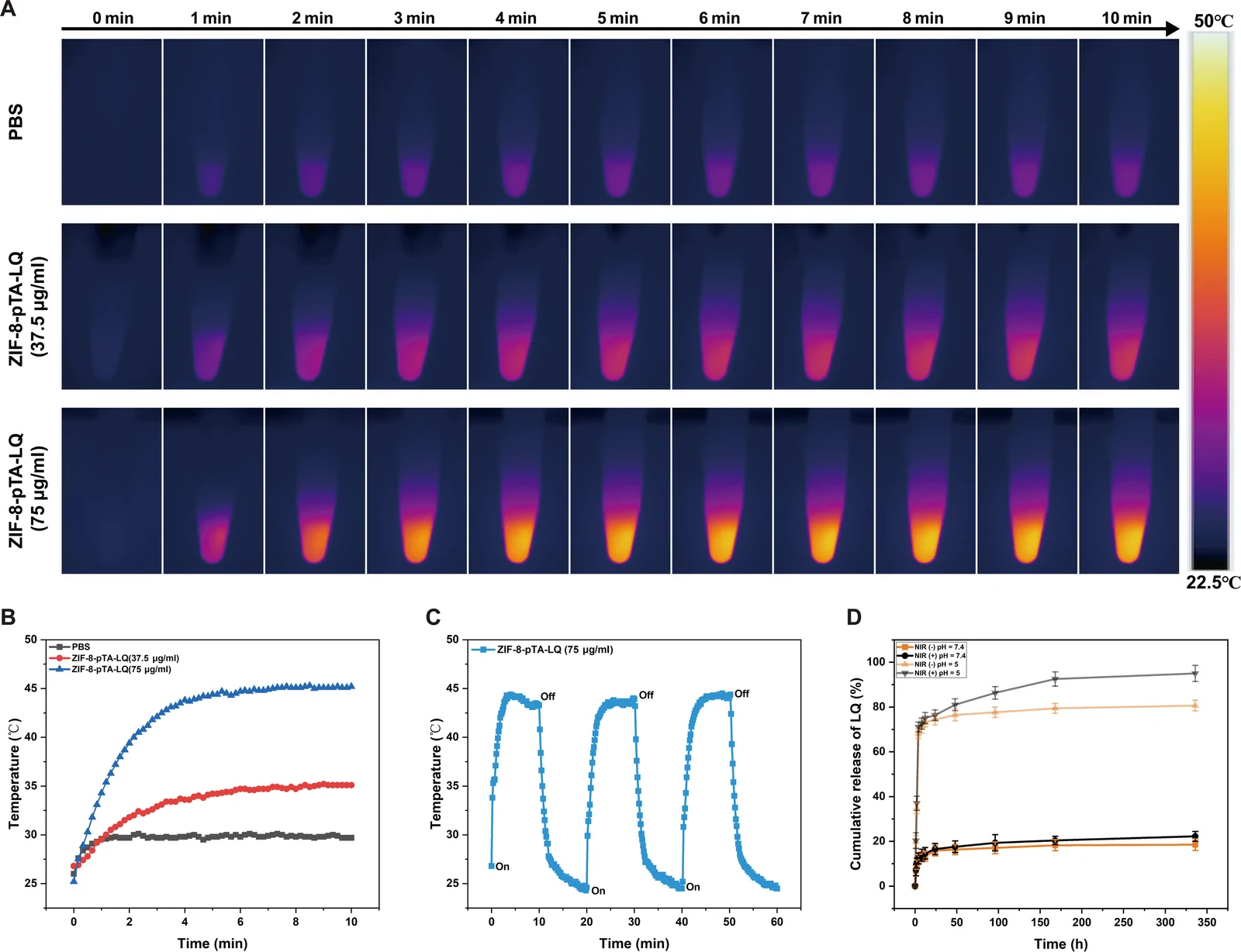
Photothermal and controlled release properties induced by NIR. (A) Thermal images of ZIF-8-pTA-LQ with gradient concentration under 808-nm NIR irradiation. (B) Temperature rise curves of ZIF-8-pTA-LQ with gradient concentration under 808-nm NIR irradiation. (C) Photothermal stability of ZIF-8-pTA-LQ under repeated on–off NIR irradiation. (D) Release properties of LQ in ZIF-8-pTA-LQ in PBS (pH 5 or 7.4) with or without NIR irradiation. The data represented means ± SD (*n* = 3). *P < 0.05, **P < 0.01, ***P < 0.001.

### Controlled release of LQ induced by NIR

The release performance of ZIF-8-pTA-LQ was detected at a concentration of 75 μg/ml determined through the abovementioned result screening. As depicted in Fig. 5D, within the initial 12 h, the release profiles demonstrated a rapid release of LQ, regardless of NIR laser irradiation and under both acidic and neutral conditions. Additionally, it is clearly demonstrated from the curves that the release of LQ in the acidic environment is more pronounced, regardless of NIR laser irradiation, with final release amounts of 95 ± 3.6% and 80.63 ± 2.36% at 14 d. In contrast, the release of LQ from this nanosystem under pH-neutral conditions was less extensive, with final release amounts of 22.2 ± 2.19% under NIR laser irradiation and 18.5 ± 2.56% without NIR laser treatment. This observation aligns with the work of Xu et al. [39] and Kong et al. [40]. The likely reason of this phenomenon is the deprotonation of the hydroxyl groups in TA at elevated pH, which results in the formation of stronger coordination bonds with the carrier’s Zn^2+^. On the other hand, regardless of whether the environment was acidic or alkaline, a clear enhancement in LQ release was observed from the nanosystem following NIR laser irradiation. This evaluation indicated that NIR irradiation on ZIF-8-pTA-LQ triggered the rise of temperature, which could prompt the release of LQ, and this phenomenon might be attributed to the thermal effect-induced attenuation of electrostatic interactions and disruption of hydrogen bonds between LQ and nanoparticles [9,10]. Besides, strengthened Brownian motion of molecules caused by rising temperatures may also be one of the reasons [41].

### Proliferation of BMSCs treated with mild hyperthermia

In order to evaluate the effect on the proliferation of BMSCs with the 808-nm NIR laser irradiating, a CCK-8 assay was used to perform the viability of cells. Obviously, the optical density (OD) value of ZIF-8-pTA and ZIF-8-pTA-LQ groups was much higher on days 1, 3, and 5, suggesting that mild temperature increase generated by photothermal effect facilitated the proliferation of BMSCs. Furthermore, statistical differences were discovered between ZIF-8-pTA and ZIF-8-pTA-LQ groups under NIR laser irradiation (Fig. 6A), which demonstrated that the released LQ from ZIF-8-pTA-LQ through NIR exposure also played a role in promoting cell proliferation. Additionally, the differences of OD value of ZIF-8-pTA-LQ with or without laser irradiation were distinct as well, implying that laser illumination accelerated the release of LQ.

**Fig. 6.**
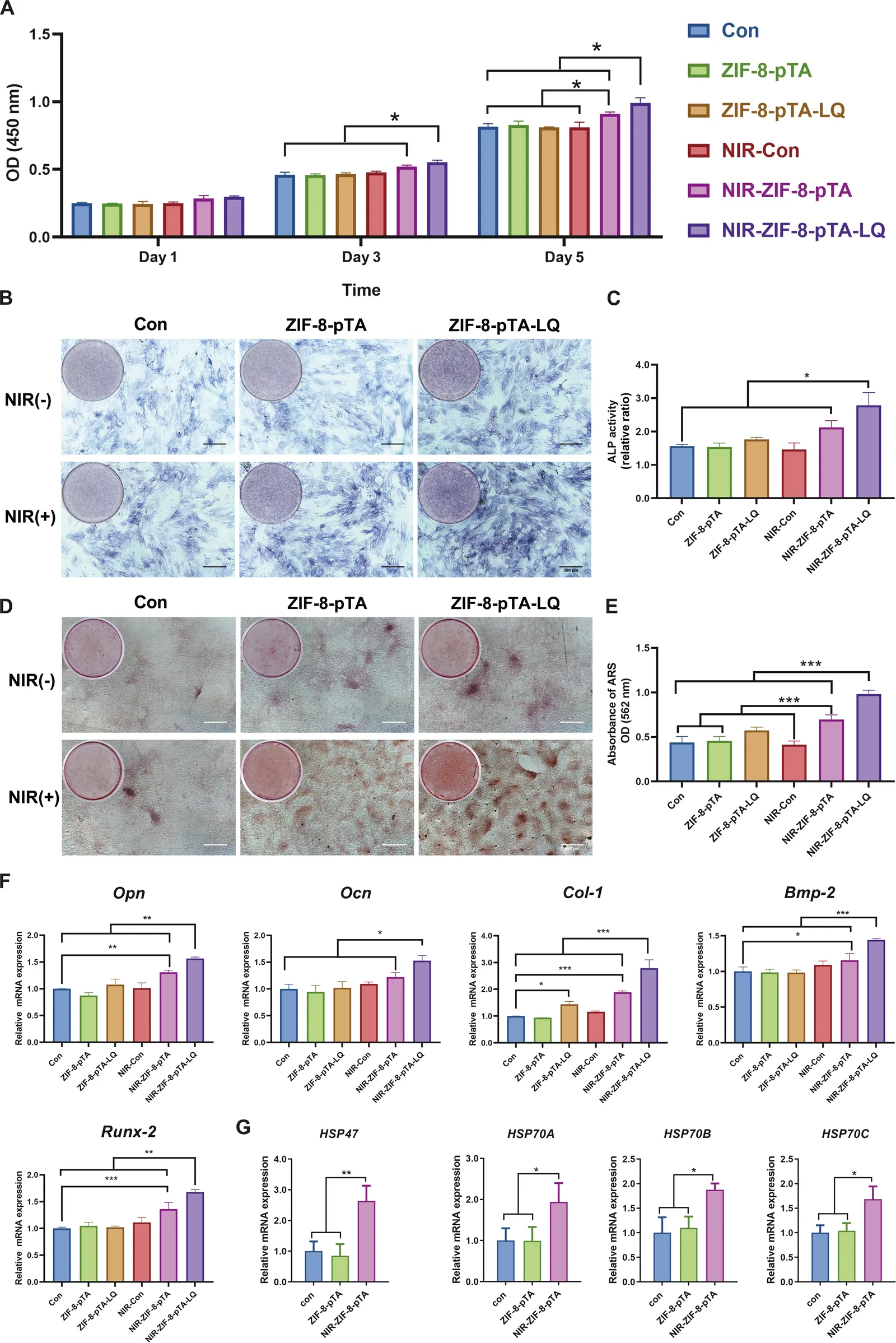
In vitro proliferation and osteogenic differentiation of BMSCs treated with mild hyperthermia. (A) Cell proliferation behavior of BMSCs with the treatment of ZIF-8-pTA and ZIF-8-pTA-LQ under NIR irradiation. The data represented means ± SD (*n* = 3). (B) ALP staining of BMSCs with the treatment of ZIF-8-pTA and ZIF-8-pTA-LQ under NIR irradiation. (C) ALP activity assay of BMSCs with the treatment of ZIF-8-pTA and ZIF-8-pTA-LQ under NIR irradiation. The data represented means ± SD (*n* = 3). (D) ARS staining of BMSCs with the treatment of ZIF-8-pTA and ZIF-8-pTA-LQ under NIR irradiation. (E) Mineralization level of BMSCs with the treatment of ZIF-8-pTA and ZIF-8-pTA-LQ under NIR irradiation. The data represented means ± SD (*n* = 3). (F) Relative mRNA expression of OPN, OCN, COL-1, BMP-2, and RUNX-2 in BMSCs with the treatment of ZIF-8-pTA and ZIF-8-pTA-LQ under NIR irradiation. The data represented means ± SD (*n* = 3). (G) Relative mRNA expression of HSPs in BMSCs with the treatment of ZIF-8-pTA under NIR irradiation. The data represented means ± SD (*n* = 3). *P < 0.05, **P < 0.01, ***P < 0.001.

### In vitro osteogenic differentiation of BMSCs treated with mild hyperthermia

To assess the osteogenic differentiation ability of BMSCs irradiated with the 808-nm NIR laser, a series of assays were conducted. ALP activity was firstly assessed with staining and qualitative methods. As shown in Fig. 6B and C, the ZIF-8-pTA-LQ group with irradiation of 808-nm NIR laser performed deepest staining compared with other groups after culturing for 7 d, followed by the ZIF-8-pTA group with laser irradiation, which demonstrated that mild hyperthermia facilitated the induction of early stage of osteogenic differentiation and the NIR-triggered LQ release from ZIF-8-pTA-LQ made a more remarkable result on this basis. Similarly, in the study by Kim et al. [42], NIR irradiation was shown to facilitate the release of BMP-2 from the nanocarrier, thereby further promoting osteogenic differentiation of pre-osteoblasts. The subsequent quantitative ALP activity assay performed corresponding tendency with above results. After 21 d of osteogenic culture, the mineralized nodules that had gradually formed were evaluated by ARS staining. The ZIF-8-pTA-LQ group with NIR irradiation performed most amounts of mineralization (Fig. 6D and E), which consistent with the results of ALP assay, implying that the temperature of 42 to 45 °C generated from NIR illumination was capable of promoting the osteogenic differentiation and accelerating the deposition of calcium mineralized nodules. In addition, several major markers for osteogenic differentiation were quantitively assessed with qRT-PCR after culturing for 7 d. In detail, osteogenic markers including RUNX2, COL-1, BMP-2, OPN, and OCN were crucial for bone regeneration, and ZIF-8-pTA and ZIF-8-pTA-LQ groups exhibited obvious up-regulation of these genes. Moreover, comparatively, the ZIF-8-pTA-LQ group demonstrated higher mRNA expression levels than the ZIF-8-pTA group (Fig. 6F). In view of the aforementioned findings, the value differences between the LQ group and the no LQ group seem to be smaller than that between the NIR and no NIR groups. This may be related to the release characteristics of LQ from the ZIF-8-pTA system under neutral culture medium conditions, as elucidated in the aforementioned explanation of the release profiles. Besides, certain amounts of investigations were involved in the regulating activity of HSPs in bone metabolism, and the promoting effect on osteogenic differentiation of MSCs by means of up-regulation of HSPs was corroborated [43,44]. In this experiment, the mRNA expression of 2 typical mammalian HSPs was examined. HSP47 is specifically responsive to heat shock and could generate unique extracellular matrix by regulating the collagen cross-linking, which is essential for the formation and maturation of osteogenesis-related type I collagen. Besides, HSP 70 distinctly enhances cell survival under thermal stress conditions by preventing protein misfolding at high temperature. Especially, HSP 70A could enhance ALP activity and promote osteogenic differentiation of BMSCs, and up-regulation of mRNA expression on these 2 HSPs in the ZIF-8-pTA group was demonstrated in Fig. 6G. In summary, the above results indicated that the mild hyperthermia produced by NIR exposure was successfully achieved, and the rising temperature made positive effects on osteogenic differentiation of BMSCs through intracellular up-regulation of HSPs and the intrinsic osteogenic function of LQ, which is released from ZIF-8-pTA-LQ upon hyperthermia induction.

### In vivo cranial defect healing of ZIF-8-pTA-LQ with NIR irradiation

Based on the previous results and analysis, the cranial defects model was utilized to investigate the healing potential of ZIF-8-pTA-LQ with NIR irradiation. In detail, rats were randomly divided into 6 groups and designated as blank, SA, ZIF-8-pTA, NIR-ZIF-8-pTA, ZIF-8-pTA-LQ, and NIR-ZIF-8-pTA-LQ. First, a clearly brighter area was observed at the cranial defect in Fig. 7A. Besides, as the real-time temperature images shown in Fig. 7B, the temperature of ZIF-8-pTA-LQ via NIR irradiation presented remarkable rise from 34.3 to 39.3 °C within 30 s and has stabilized at around 41 °C after 2 min of NIR treatment. In comparison, the cranial defect with restoration of SA exhibited temperature increase merely from 35.3 to 37.3 °C. Eventually, this result confirms that the NIR irradiation satisfied the ability to constantly penetrate through tissue barrier and maintains relatively high thermal conversion efficiency.

**Fig. 7.**
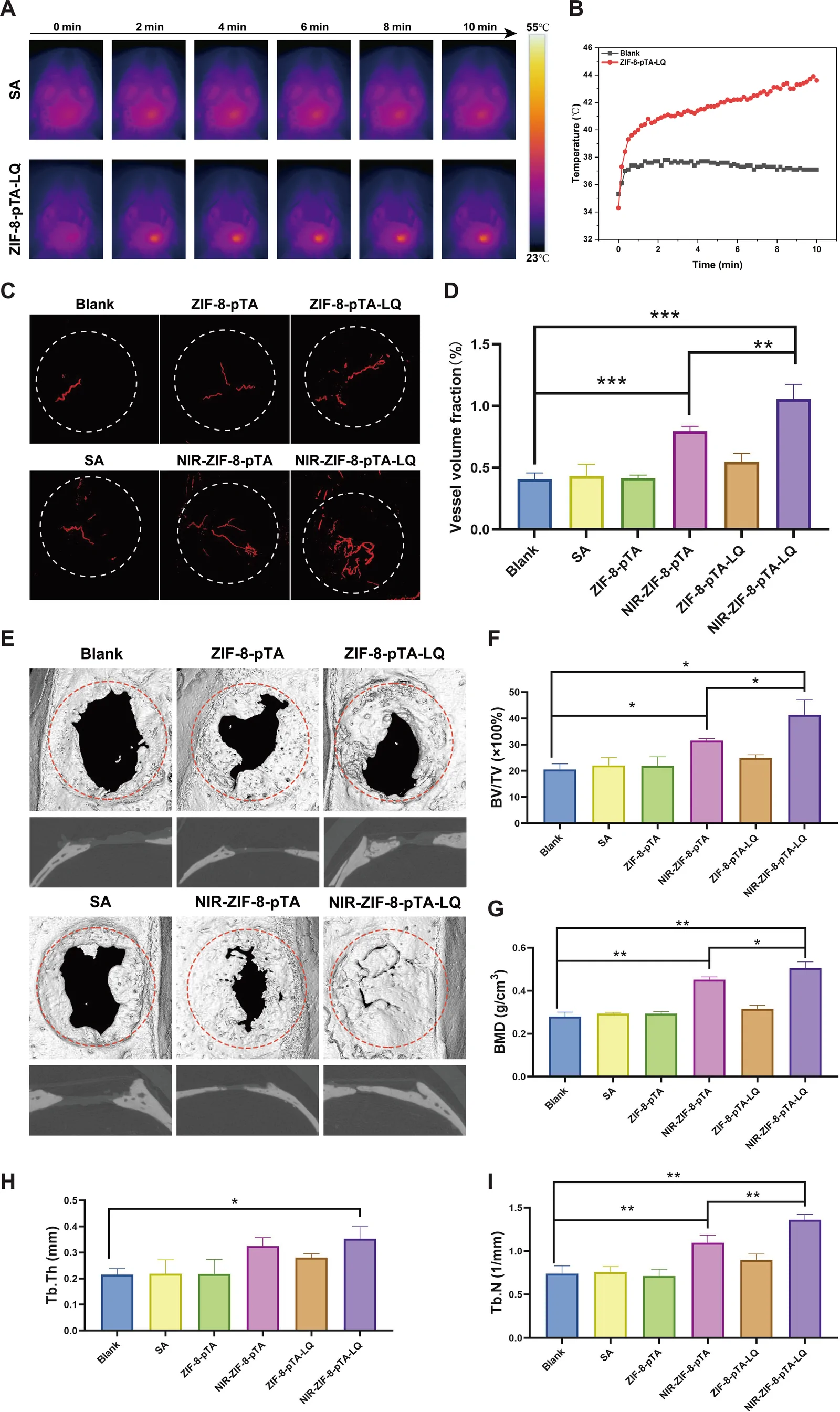
Photothermal properties and cranial defect healing in vivo. (A) Infrared thermographic photographs of ZIF-8-pTA-LQ with NIR irradiation in vivo. (B) Temperature rise curves of ZIF-8-pTA-LQ with NIR irradiation in vivo. (C) Micro-CT images reconstructed after Microfil perfusion and explantation at the cranial defect area with different hydrogel implanting. (D) Quantitative analysis of blood vessel volume fraction. The data represented means ± SD (*n* = 3). (E) Micro-CT images of bone repair in the blank, SA, ZIF-8-pTA, NIR-ZIF-8-pTA, ZIF-8-pTA-LQ, and NIR-ZIF-8-pTA-LQ groups. (F to I) Quantitative analysis of micro-CT results, including BV/TV, BMD, Tb.Th, and Tb.N. The data represented means ± SD (*n* = 3). *P < 0.05, **P < 0.01, ***P < 0.001.

After 4 weeks of surgery, half of the rats were perfused with Microfil MV-120 solution and curing agent and the samples were harvested and then scanned with micro-CT to evaluate vascularization in the cranial defects. As shown in Fig. 7C, the newly formed vessels in the defect area were observed. Both NIR-ZIF-8-pTA-LQ and NIR-ZIF-8-pTA groups exhibited larger vessel volume fraction than the other groups. In addition, the former samples showed more microvessel ingrowth within the defect area than did the NIR-ZIF-8-pTA group (Fig. 7D). The other half samples harvested from rats humanely euthanized at 8 weeks after operation were directly scanned by micro-CT to assess the formation of new bone at the defected craniums. Three-dimensional CT reconstruction images and cross-sections of each group are shown in Fig. 7E. The NIR-ZIF-8-pTA-LQ group presented the marked bone regeneration effects, shown at a ratio of new bone volume/total volume (BV/TV) of 41.41%, which was higher than the other groups. The bone mineral density (BMD), trabecular thickness (Tb.Th), and number of bone trabecula (Tb.N) were also analyzed and indicated nearly the same tendency as BV/TV. Additionally, the NIR-ZIF-8-pTA group also had superior BV/TV, BMD, and Tb.Th than the blank group (Fig. 7F to I). Combined with the above mentioned analysis, the mild heat stimulation generated from NIR irradiation on ZIF-8-pTA could accelerate vascularization, which was crucial for the bone repair [45]. Corresponding results of new bone formation were observed. These findings potentially ascribed to up-regulation of HSPs triggered by hyperthermia. In addition, the difference between NIR-ZIF-8-pTA-LQ and NIR-ZIF-8-pTA in promoting angiogenesis and bone repair can be attributed to the PI3K/Akt pathway of BMSCs induced by LQ. However, the value differences of degree of bone repair between the ZIF-8-pTA group and the ZIF-8-pTA-LQ group seem to be smaller than those between the NIR- ZIF-8-pTA and ZIF-8-pTA groups. I was noted that the release amount of LQ was comparatively low in the neutral pH environment. Although a mildly acidic environment forms after bone defect, the pH is nevertheless relatively high and transient [46]. Consequently, the level of LQ released is likely insufficient to prevent the phenomenon. Therefore, future work should focus on identifying more suitable drug molecules for this nanosystem. The synergistic effect between LQ and photothermal therapy in bone regeneration has thereby been demonstrated.

Mineralization of newly formed bone was visualized by sequentially injecting into rats with calcein (green) and alizarin red (red). As shown in Fig. 8A, the fluorescent labeling areas of NIR-ZIF-8-pTA-LQ were remarkably larger than those in other groups (Fig. 8B). Additionally, as shown in Fig. S3, the expression of the inflammatory cytokine TNF-α was consistent across all groups, and no signs of obvious pathological changes in the major organs of rats receiving any of the nanoparticle treatments were observed in Fig. S4. The histological analysis by H&E and Masson’s trichrome staining of cranium tissue sections was illustrated in Fig. 8C. Judging from the H&E staining results, the defect area is mainly composed of fibrous connective tissue in the blank, SA, and ZIF-8-pTA groups, and segmental newly formed bone can be observed in the NIR-ZIF-8-pTA and ZIF-8-pTA-LQ groups. The bone tissue regenerated from the periphery to the center of the defect is almost completely integrated in the NIR-ZIF-8-pTA-LQ group. Masson’s trichrome staining is able to dye collagen fibers in the bone matrix, with immature bone appearing predominantly blue and mature bone staining red. In the NIR-ZIF-8-pTA-LQ group, continuous and abundant blue with a few red bone collagen fibers were observed, suggesting active bone regeneration coupled with mineralization processes within the defect area. Further immunofluorescence costaining was conducted using osteogenic marker OPN (green) and vascular endothelial marker CD31 (red) to assess local osteogenesis and angiogenesis within the defect area. As shown in Fig. 8D and E, both NIR-ZIF-8-pTA-LQ and NIR-ZIF-8-pTA groups subjected to NIR irradiation-induced photothermal treatment demonstrated up-regulated expression of OPN and CD31, with the LQ-modified group exhibiting higher expression levels. This result is consistent with the performance of micro-CT analysis and histological staining images. To further validate the mechanism of function of NIR-ZIF-8-pTA-LQ in promoting cranial bone repair in rats, the immunohistochemical staining and corresponding quantitative analysis were carried out to evaluate the expression level of HSP47 and p-Akt. Figures S5 and S6 demonstrated that the NIR-ZIF-8-pTA and NIR-ZIF-8-pTA-LQ groups exhibited a larger area of HSP47 expression (7.72 ± 1.10% and 7.44 ± 1.42%, respectively) along with more intense staining relative to the other groups. Regarding p-Akt, a broader expression area was detected in the NIR-ZIF-8-pTA-LQ group. Looking to the future, NIR light can penetrate the thin mucosal layer and reach the implant site, where it generates PTT for repairing mandibular defects. This approach also remains highly beneficial for managing exposed or infected bone defects [47–49], indicating substantial promise for clinical translation.

**Fig. 8.**
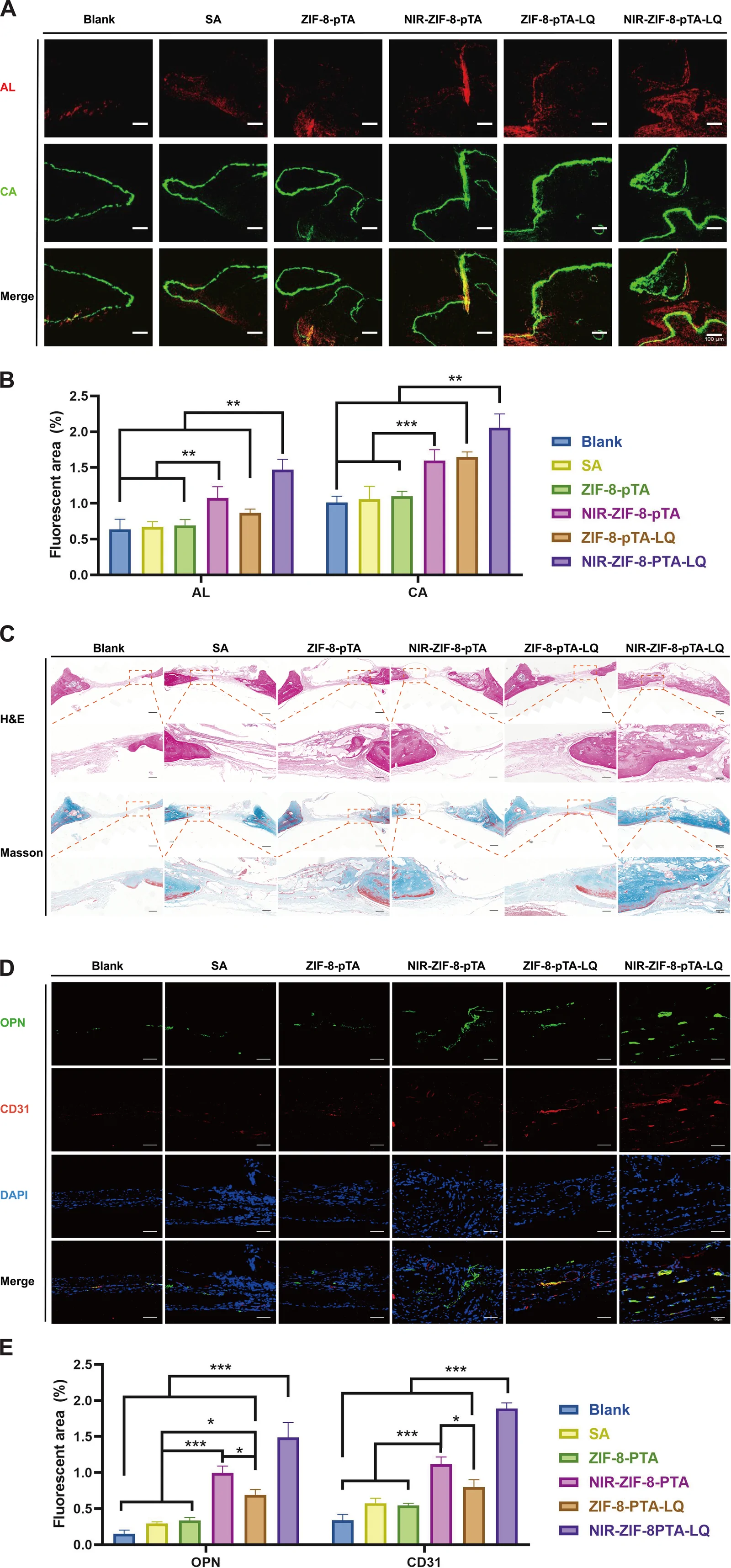
Histological and histomorphometric observation*.* (A) Confocal images of hard tissue sections after sequential fluorescence labeling, namely, alizarin (AL) and calcein (CA). (B) Quantitative analysis was based on 3 randomly selected high-power fields. The data represented means ± SD (*n* = 3). (C) H&E and Masson staining on week 8. (D) Immunostaining of OPN and CD31 on week 4. (E) Quantitative analysis was based on 3 randomly selected high-power fields. The data represented means ± SD (*n* = 3). *P < 0.05, **P < 0.01, ***P < 0.001.

## Conclusion

In summary, photothermal ZIF-8-pTA-LQ nanoparticles with controlled release of LQ are successfully fabricated. The temperature (42 to 45 °C) that could up-regulate the HSPs generated from the NIR irradiation with efficient photothermal conversion in vitro, and with favorable penetration of NIR through tissue barrier, sustaining local appropriate heating, was achieved. The experimental results also revealed the NIR irradiation-induced release of LQ, and with combination of the activation in the PI3K/Akt pathway by LQ and the up-regulation of mRNA expression of HSPs, the NIR-ZIF-8-pTA-LQ group demonstrated outstanding performance in vascularized osteogenesis. This photothermal methodology with convenience and efficiency exhibits substantial potential for the treatment of bone defect cases in certain clinical scenarios.

## Data Availability

Data will be made available on request.
