# Supplementary material for: Near-Infrared Light-Responsive Nanosystems with Controlled l-Quebrachitol Release and Photothermal Effect for Synergistic Bone Defect Regeneration
Source: Biomater Res. 2026 Oct 30;30:0357. doi: 10.34133/bmr.0357 (PMC13720139; doi:10.34133/bmr.0357)
Supplement: Supplementary file 1 [file bmr.0357.f1.docx]

Supporting Information

**Near-infrared light-responsive nanosystems with controlled L-quebrachitol release and photothermal effect for synergistically bone defect regeneration**

Zhengkuan Lu^1,2†^, Lingyan Cao^3†^, Shuaiting Wu^4^, Ao zheng^3^, Shuxian Lin^2^*, Jiansheng Su^1^*

**Fig. S1.** Cell viability of BMSCs after treatment with gradient concentration of ZIF-8-pTA. The data represented means ± SD (n = 3).

**Fig. S2.** Cell viability of BMSCs after treatment with gradient concentration of ZIF-8-pTA-LQ. The data represented means ± SD (n = 3).

**Fig. S3.** Immumohistochemical staining of TNF-α on week 4.

**F****ig. S4.** HE staining images of major organs (including heart, liver, spleen, lung and kidney) of different groups.

**Fig. S5.** Immumohistochemical staining of HSP47 and p-Akt on week 4.

**Fig. S6.** Quantitative analysis of immumohistochemical staining area coverage of HSP47 and p-Akt. The data represented means ± SD (n = 3). (** denotes *p* < 0.01, *** denotes *p* < 0.001)

**Table S1**. Gene primer sequences for RT-PCR.

| Gene | | Forward primer (5' - 3') | Reverse primer (5' - 3') | |
| --- | --- | --- | --- | --- |
| **ALP**  **OCN**  **OPN**  **RUNX2**  **COL-1**  **VEGF**  **GSK-3β**  **β-catenin**  **BMP-2**  **HSP 47**  **HSP 70A**  **HSP 70B**  **HSP 70C**  **GAPDH** | TATGTCTGGAACCGCACTGAAC  GCCCTGACTGCATTCTGCCTCT  CCAAGCGTGGAAACACACAGCC  ATCCAGCCACCTTCACTTACACC  CAGGCTGGTGTGATGGGATT  GGCTCTGAAACCATGAACTTTCT  ACCATCCTTATCCCTCCTCAC  AAGGTGCTGTCTGTCTGCTCTA  GTCTTCTAGTGTTGCTGCTTCCC  GCTGCTCGTCAACGCCATGT  TGTTCCGTTTCCAGCCCCCAA  CTCCAGCATCCGACAAGAAGC  TTGAGGAGGTGGATTAGGGGC  CTGGAGAAACCTGCCAAGTATG | | | CACTAGCAAGAAGAAGCCTTTGG  TCACCACCTTACTGCCCTCCTG  GGCTTTGGAACTCGCCTGACTG  GGGACCATTGGGAACTGATAGG  CCAAGGTCTCCAGGAACACC  GCAATAGCTGCGCTGGTAGAC  TTATTGGTCTGTCCACGGTCT  CTTCCATCCCTTCCTGCTTAGT  TCTCTGCTTCAGGCCAAACAT CCATCCAGGTCTTCAGCTGC  TAGTTGCTGCCTCTGTTCGGG  GGACTTGGGGGTGTTGTGGCA  CCGCTTTTGTGATGTTTCCGA  GGTGGAAGAATGGGAGTTGCT |
